# Supplementary material for: Neural mechanisms of flexible perceptual inference
Source: PLoS Comput Biol. 2025 Dec 1;21(12):e1013675. doi: 10.1371/journal.pcbi.1013675 (PMC12680366; doi:10.1371/journal.pcbi.1013675)
Supplement: S1 Text — (PDF) [file pcbi.1013675.s001.pdf]

Neural mechanisms of flexible perceptual inference
S1 Text

John Schwarcz[1◑], Jan Bauer[1,2◑], Haneen Rajabi[1], Gabrielle Marmur[1], Robert Reiner[1], Eran Lottem[1‡], Jonathan Kadmon[1‡*],

1 Edmond and Lily Safra Center for Brain Sciences (ELSC), The Hebrew University of Jerusalem, Jerusalem, Israel
2 Gatsby Computational Neuroscience Unit, University College London, London, United Kingdom

◑These authors contributed equally to this work.
‡These authors contributed equally to this work.
* jonathan.kadmon@mail.huji.ac.il

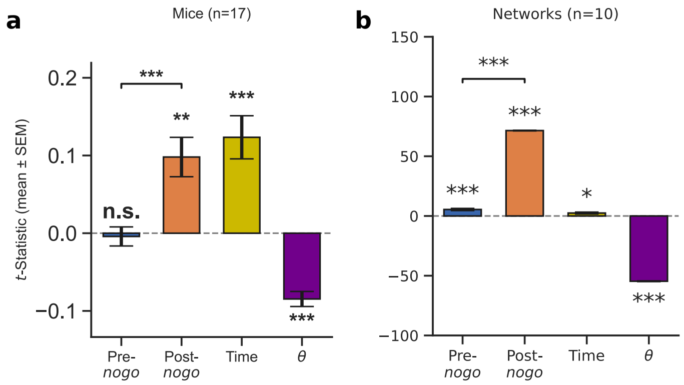

Fig S1. *GLM analysis of mice **(a)** and trained networks **(b)**.* We performed a generalized linear model (GLM) analysis with a logit link function to test whether the evidence accumulation underlying mouse and network behavior exhibits nonlinear resetting in response to *nogo* cues (with mixed effects included for mice). The dependent variable was the probability of acting ($\in [0,1]$) in each time bin (50 ms bins for mice; 1 timestep for networks), up to and including the time of action. The model included four independent variables: *Pre-nogo*, *Post-nogo*, *Time*, and θ. *Pre-nogo* is defined in each bin as the number of *go* cues that occurred prior to the most recent *nogo* (set to 0 before the first *nogo*). *Post-nogo* is defined as 0 during a *nogo*, and otherwise the number of consecutive *go* cues since the most recent *nogo*. These variables capture evidence accumulation without (*Pre-nogo*) and with (*Post-nogo*) resetting. *Time* within the trial and the contextual parameter θ were included to control for unrelated sources of variability. Plotted on the y-axis are group-averaged t-statistics (mean $\pm$ standard error) for each regressor, along with the results of one-sample t-tests against a null hypothesis of zero. A paired two-tailed t-test comparing the *Pre-nogo* and *Post-nogo* coefficients revealed a significant difference, supporting nonlinear resetting.

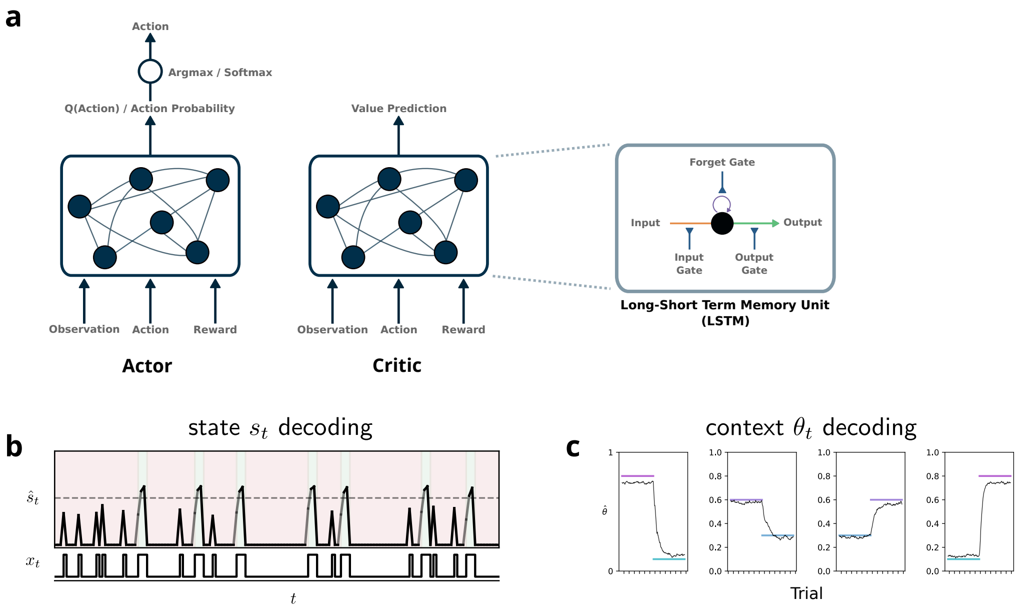

Fig S2. *Network architecture and task-variable decoding.* **(a)** Actor-critic and LSTM architecture. Actor and critic were each comprised of a Pytorch LSTM. **(b)** State decoding for example stimulus trajectory. Background color indicates ground truth s_t. Dashed horizontal line indicates $\hat{s}_t = 0.5$ and line thickness corresponds to estimate accuracy. **(c)** Context decoding for example block transitions (averaged over several transitions). Colored lines indicate ground truth θ_t.

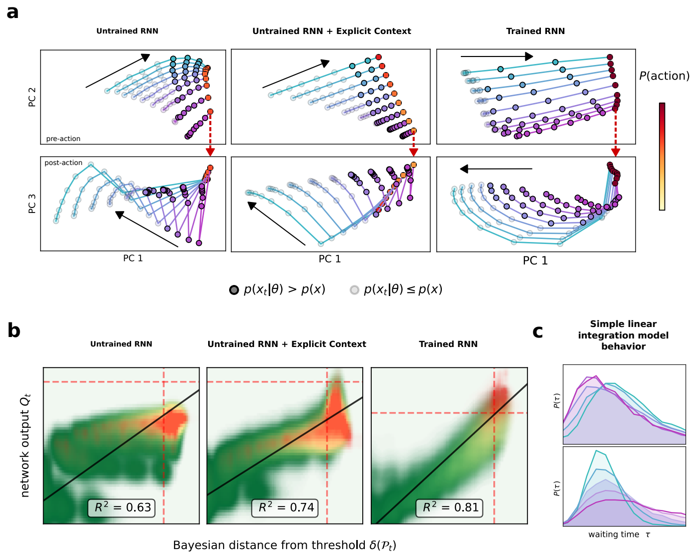

Fig S3. *Network representations.* **(a)** Projection of network activity onto the first three principal components (PCs) for trained and untrained networks (with and without explicit context). Top: Pre-action trajectories in PC1–PC2 space for the 9 timesteps leading up to action. Bottom: Post-action trajectories in PC1–PC3 space for the 9 timesteps following action. Top and Bottom panels are arranged such that trial start to trial end follows a clockwise path. Colors of action-time circles indicate the average probability of action $\langle p(\text{action})\rangle_{t|\theta}$ generated by the readout at the moment of action. Training reshapes typical trajectories such that all θ values span a similar dynamic range during the *go* sequence. In contrast, untrained networks exhibit compressed representations in high θ due to the reduced variance in the stimulus. While explicit context allows the network to produce a context-dependent peak in action probability, the representation remains entangled—preventing the readout from implementing the optimal policy. **(b)** Heatmaps of Q_t versus the Bayesian decision variable δ_t during *go* cues ($x_t = 1$) for each network. In untrained networks, Q_t saturates before crossing 0, limiting policy expressiveness. In trained networks, Q_t closely tracks $\delta(\mathcal{P}_t)$ and crosses 0 for all values of θ, supporting a deterministic policy. **(c)** A simple linear integration model replicates key behaviors of untrained networks. Top: Model behavior with no context dependence ($\epsilon_\theta = 0$; $\alpha = 0.4$, $\beta = 6$, $\epsilon_0 = -7.3$). Bottom: Model behavior with context-dependent bias ($\epsilon_\theta = -3\theta$; $\alpha = 0.4$, $\beta = 10$, $\epsilon_0 = -9.4$).

S4. Detailed analytical derivation of the Bayes-optimal inference and policy

Closed-form solution for reward rate in fixed context

Determining the optimal strategy for action requires estimating $P(s_t|\boldsymbol{x}_{\leq t})$ and thresholding on its value. Because $x_t = 0$ implies $s_t = 0$, it is sufficient to count cues from the last negative observation $x_{t-\tau} = 0$, i.e.
$P(s_t|\boldsymbol{x}_{\leq t}) = P(s_t|\boldsymbol{x}_{t-\tau:t} = 1,\, x_{t-\tau} = 0)$, where we use the slicing notation $t-\tau : t$ to mean all times t' fulfilling $t - \tau < t' \leq t$ (Matlab-style indexing). The waiting time τ hence forms the central component in the actor's policy.

In the case where cues are faithful estimates of the latent state, $\mathbb{E}\left[x_t\right] = s_t$, their summed number will be increasing evidence that the latent state has switched. This idea has been formalized in sequential decision making, where the log-odds ratio $\log \frac{P(s_t=1|\boldsymbol{x}_{\leq t})}{P(s_t=0|\boldsymbol{x}_{\leq t})}$ gets updated adaptively. In our setting, this translates to $\Delta \log \hat{s} \simeq \log \theta_t^{-1}$ Herein, the inverse likelihood $\theta_t^{-1} = P(x_t = 1|s_t = 0, \theta_t)^{-1}$ appears as the slope of the integrator, i.e., the reliability of the cues.

We further define auxiliary variables

$$b \equiv P(x_t = 1, s_t = 0|s_{t-1} = 0,\, \theta) = \theta\,(1-\lambda)$$
$$c \equiv P(x_t = 0, s_t = 0|s_{t-1} = 0,\, \theta) = (1-\theta)\,(1-\lambda)$$

Transition after τ observations. In order to evaluate the reward rate in the main text, we need to calculate $P(s_t = 1|\boldsymbol{x}_{\leq t},\, \theta)$. Because an unsafe cue $x_t = 0$ always implies the unsafe state, it is sufficient to calculate $P_{s=1} \equiv P(s_t = 1|\boldsymbol{x}_{t-\tau:t} = 1,\, \theta)$, i.e. how likely the safe state is after τ suggestive observations, where we introduced $P_{s=1}$ for brevity. To this end, we consider the complement $P_{s=0} = 1 - P_{s=1}$. To evaluate this expression, we formulate a self-consistent relation,

$$P_{s=0} = b^\tau + \sum_{k=0}^{\tau-1} b^k c\, P_{s=0}$$
$$\Rightarrow P_{s=0} = \frac{b^\tau}{1 - \sum_{k=0}^{\tau-1} b^k c}.$$

This equation considers the probability b^τ to have been unsafe the entire time. On top of this, the second sum considers all worlds where k observations $x = 1$ where followed by a faithful negative $x = 0$. By the Markov property of the world, these paths again reweight $P_{s=0}$ itself. Resubstitution finally yields $P_{s=1} = 1 - P_{s=0}$. To cross-check this relation, we simulated it via Monte Carlo sampling of the world.

Expected reward probability and action time The average reward when waiting for τ steps before acting is given by the probability the agent is acting in a safe state, $R = P(s_t = 1|\theta, \boldsymbol{x}_{t-\tau:t} = 1)$. The probability of being safe can be written explicitly as

$$R(\tau;\theta) = P\left(s_t = 1|\theta, \boldsymbol{x}_{t-\tau:t} = 1\right) = 1 - \frac{b^\tau}{1 - c\sum_{k=0}^{\tau-1} b^k}, \tag{1}$$

where $b \equiv (1-\lambda)\theta$ is the probability of a misleading *go* (i.e., the probability the state did not change times the probability of it being flipped), and $c \equiv (1-\lambda)(1-\theta)$ is the probability of observing a *nogo*.

To arrive at the final expression for the reward rate, the average time to reach τ cues is required, as well. We here extend the calculation presented by [1] for a standalone binary variable with the latent context in our setting.

Let $\bar{T} \equiv \mathbb{E}\left[T \mid \boldsymbol{x}_{t-\tau:t} = 1, \theta\right]$ denote the expected action time, where τ indicates the period of consecutive cues $x_{t-\tau:t} = 1$ preceding any action. Oftentimes it is possible to solve such problems by trying to write a recursive relation that describes how the quantity of interested (here $\bar{T}$) relates from one time step to another and then solve for it [1]. This gives

$$\bar{T}(\tau;\theta) = b^{\tau}\tau + \sum_{k=0}^{\tau-1} b^k \lambda \tau \cdot 1 + b^k c \left(\bar{T}(\tau,\theta) + k + 1\right).$$

Here, we have summed up all possible paths to get τ consecutive $x_{t-\tau:t} = 1$: The first term is the probability of getting τ *unsafe* cues in $s_{t-\tau:t} = 0$ right away, as it is just the τth power of the probability b of a misleading go cue. The sum in the second term then sums up the $k = 0 \ldots \tau - 1$ different paths where each path is defined by having had exactly k many unsafe cues $x_t = 1|s_t = 0$. From there on, a transition to *safe* happens with probability λ, and all subsequent cues in this trial will be guaranteed to be faithfully *safe*, yielding the factor 1. The second term starts out with the same b^k, but then captures the paths where with probability c, the world does not transition to *safe*. Again because of the Markovian structure, the world statistically indifferent from the initial time. Therefore, agent has to wait for $\bar{T}(\tau;\theta)$ on expectation again, in addition to the cost $k+1$ that it lasted to get to this point, with $+1$ accounting for the negative cue. This is the key step that reproduces the left-hand side and allows us to solve the equation.

Rearranging for $\bar{T}$, we then obtain

$$\bar{T}(\tau;\theta) = \frac{b^{\tau}\tau + \sum_{k=0}^{\tau-1} b^k \lambda \tau + b^k c\,(k+1)}{1 - \sum_{k=0}^{\tau-1} b^k c}. \tag{2}$$

From this, the reward rate becomes

$$\mathbb{E}\left[r_t | \boldsymbol{x}_{\leq t},\ \theta\right](\tau) = \frac{1}{\bar{T}(\tau,\theta)} P(s_t = 1 | \boldsymbol{x}_{\leq t},\ \theta) = \frac{1}{\bar{T}(\tau,\ \theta)} P(s_t = 1 | \boldsymbol{x}_{t-\tau:t} = 1,\ \theta).$$

The maximizer $\tau^{\star}$ of this expression defines the optimal policy in a context θ. Incorporating the *premature* state yields the modified expression:

$$\mathbb{E}\left[r_t \mid \boldsymbol{x}_{\leq t},\ \theta\right](\tau) = \frac{P(s_t = 1 \mid \boldsymbol{x}_{t-\tau:t} = 1,\ \theta)}{\bar{T}(\tau,\ \theta) + T_{\text{prem}}\big(1 - P(s_t = 1 \mid \boldsymbol{x}_{t-\tau:t} = 1,\ \theta)\big)}.$$

Here, T_{prem} denotes the duration of the *premature* state. Since this state was neither necessary for nor did it significantly affect any results, it was omitted from theoretical and simulation analyses for simplicity.

Latent variables require tracking joint density

In this section, we show that updating of the marginals of the joint belief cannot be done without holding track of the full joint distribution at the previous time step. This necessitates using the full joint update equation in the main text.

State

$$
\begin{aligned}
P(s_t|\boldsymbol{x}_{\leq t} = (x_t, \boldsymbol{x}_{<t})) &\propto P(s_t, x_t|\boldsymbol{x}_{<t}) \qquad (3)\\
&= \sum_{\theta_t} P(s_t, x_t, \theta_t|\boldsymbol{x}_{<t})\\
&= \sum_{\theta_t} P(x_t, \theta_t|s_t, \boldsymbol{x}_{<t}) P(s_t|\boldsymbol{x}_{<t})\\
&= \sum_{\theta_t} P(x_t|s_t, \theta_t) P(\theta_t|s_t, \boldsymbol{x}_{<t}) P(s_t|\boldsymbol{x}_{<t})\\
\text{def. conditional} &= \sum_{\theta_t} P(x_t|s_t, \theta_t) \frac{P(s_t, \theta_t|\boldsymbol{x}_{<t})}{P(s_t|\boldsymbol{x}_{<t})} P(s_t|\boldsymbol{x}_{<t}).\\
&= \sum_{\theta_t} P(x_t|s_t, \theta_t) P(s_t, \theta_t|\boldsymbol{x}_{<t})\\
&= \sum_{\theta_t} P(x_t|s_t, \theta_t) \sum_{s'_{t-1}} \boldsymbol{T}_{s_t s'_{t-1}} \sum_{\theta'_{t-1}} \boldsymbol{T}_{\theta_t \theta'_{t-1}} P_{t-1}(s'_{t-1}, \theta'{}_{t-1}|\boldsymbol{x}_{<t}).
\end{aligned}
$$

Importantly, in the third line, $P(s_t|\theta_t, \boldsymbol{x}_{<t}) \neq P(s_t|\boldsymbol{x}_{<t})$, in general. This can be seen from the fact that high noise will make the safe state more unlikely.

Context This follows completely analogously

$$
\begin{aligned}
P(\theta_t|\boldsymbol{x}_{\leq t} = (x_t, \boldsymbol{x}_{<t})) &\propto P(\theta_t, x_t|\boldsymbol{x}_{<t}) \qquad (4)\\
&= \sum_{s_t} P(s_t, x_t, \theta_t|\boldsymbol{x}_{<t})\\
&= \sum_{s_t} P(s_t, x_t|\theta_t, \boldsymbol{x}_{<t}) P(\theta_t|\boldsymbol{x}_{<t})\\
&= \sum_{s_t} P(x_t|s_t, \theta_t) P(s_t|\theta_t, \boldsymbol{x}_{<t}) P(\theta_t|\boldsymbol{x}_{<t})\\
\text{def. conditional} &= \sum_{s_t} P(x_t|s_t, \theta_t) \frac{P(s_t, \theta_t|\boldsymbol{x}_{<t})}{P(\theta_t|\boldsymbol{x}_{<t})} P(\theta_t|\boldsymbol{x}_{<t})\\
&= \sum_{s_t} P(x_t|s_t, \theta_t) P(s_t, \theta_t|\boldsymbol{x}_{<t})\\
&= \sum_{s_t} P(x_t|s_t, \theta_t) \sum_{s'_{t-1}} \boldsymbol{T}_{s_t s'_{t-1}} \sum_{\theta'_{t-1}} \boldsymbol{T}_{\theta_t \theta'_{t-1}} P_{t-1}(s'_{t-1}, \theta'{}_{t-1}|\boldsymbol{x}_{<t}).
\end{aligned}
$$

Note that these relations are completely equivalent to each other, up to a normalization.

In summary, this shows that the update of the marginals necessitates keeping track of the joint priors. Intuitively, this comes about in line 3, where the belief about the *joint* occurrence of s_t, θ_t enters. Note that we can recover these relations by marginalization of the joint update in the main text, for example for the s_t update

$$
\begin{aligned}
P(s_t|\boldsymbol{x}_{\leq t}) &= \sum_{\theta_t} P(s_t, \theta_t|\boldsymbol{x}_{\leq t})\\
&= \sum_{\theta_t} P(x_t|s_t, \theta_t) \sum_{s'_{t-1}} \boldsymbol{T}_{s_t s'_{t-1}} \sum_{\theta'_{t-1}} \boldsymbol{T}_{\theta_t \theta'_{t-1}} P_{t-1}(s'_{t-1}, \theta'{}_{t-1}|\boldsymbol{x}_{<t}).
\end{aligned}
$$

where we used the normalization of the transition matrix and the prior in the last two steps. This goes likewise for $P(\theta_t|\boldsymbol{x}_{\leq t})$. The state and context estimators of the Bayesian agent were computed as averages with respect to the updated joint distribution.

Bayesian inference without approximations

Because the transition functions were time-dependent, we made several approximations to make the inference process Markovian. The time-dependent transition functions without any approximations are as follows:

Time dependent ITI transition probabilities could be modeled as in [2] with microstates (denoted as $s = k$ for $k \in [-u_{\max}, ..., -1]$) where $u_{\min}$ and $u_{\max}$ are the minimum and maximum ITI durations. For each microstate the transition probability from ITI to unsafe is given by:

$$P(s_t = 0|s_{t-1} = k) = \begin{cases} 0, & \text{if } |k| > u_{\max} - u_{\min} \\ |k^{-1}|, & \text{otherwise} \end{cases}$$

and the probability of transitioning to the next ITI microstate is simply given by

$$P(s_t = k+1|s_{t-1} = k) = 1 - P(s_t = 0|s_{t-1} = k)$$

The fact that θ only changes at the end of an ITI is fully captured by:

$$P(\theta_t, s_t|\theta_{t-1}, s_{t-1} = -1) = \begin{cases} 1 - B^{-1}, & \text{if } \theta_t = \theta_{t-1} \\ B^{-1}/(|m| - 1), & \text{otherwise} \end{cases}$$

where B is the length of a block of constant θ and

$$P(\theta_t, s_t|\theta_{t-1}, s_{t-1} \neq -1) = P(\theta_t, s_t|\theta_{t-1}, s_{t-1} \neq -1) = \begin{cases} 1, & \text{if } \theta_t = \theta_{t-1} \\ 0, & \text{otherwise} \end{cases}$$

For the results in the main text we set $u_{\min} = u_{\max} = 1$ and approximated context transitions with stationary transition probabilities. This did not effect the performance of the Bayesian agent which remained near-optimal under both approximations.

References

1. Ginsparg P. Lecture notes on mathematical methods for information science. INFO295. 2005;.

2. Hennig J, Romero Pinto SA, Yamaguchi T, Linderman SW, Uchida N, Gershman SJ. Emergence of belief-like representations through reinforcement learning. bioRxiv : the preprint server for biology. 2023; p. 2023–04.
