# Supplementary material for: Neural mechanisms of flexible perceptual inference
Source: PLoS Comput Biol. 2025 Dec 1;21(12):e1013675. doi: 10.1371/journal.pcbi.1013675 (PMC12680366; doi:10.1371/journal.pcbi.1013675)
Supplement: Attachment — Submitted filename: Detailed response to reviewers.pdf [file pcbi.1013675.s002.pdf]

Summary of main changes

We thank the reviewers and the editor for their careful reading of our manuscript and for their constructive and insightful comments. We appreciate the generally positive and supportive tone of the reviews, as well as the important questions raised. In response, we have revised the manuscript to address these points and believe that the additions and clarifications have sharpened both the message and the conclusions of the work. Below, we first summarize the major changes made in response to comments from multiple reviewers and the editor. We then address each reviewer comment in detail. Minor points and typographical errors have all been corrected and are not discussed further.

More detailed comparison to a standard drift-diffusion model (DDM)

A central concern raised by the reviewers and editor is the need to compare our framework to standard DDMs. We wish to emphasize two key points. First, our Bayesian inference framework is not an alternative or competing model to DDM, but rather provides the normative optimal solution under uncertainty about context and input reliability. If the context is known, the classic DDM (with the correct integration rate or threshold) is indeed optimal. The real challenge addressed in our work is optimal inference of the context itself and its neural implementation.

Second, this distinction makes direct comparison to a traditional DDM ill-posed: the Bayesian solution formalizes the best possible behavior, while the DDM assumes context is already given. For a meaningful baseline, we therefore compare to a simple leaky linear integrator, which attempts to infer context by averaging recent evidence (e.g., Usher & McClelland 2001) . This approach represents the most straightforward extension of DDM ideas to the problem of context inference. This model holds independent state and context estimates, in contrast to the Bayesian joint inference.

To clarify these points, **we have added a new subsection** (3.3, “Tractable approximations”) directly comparing the leaky DDM to the Bayes-optimal framework. We show that the leaky integrator is suboptimal, especially in its inability to jointly optimize reward rate and adaptation speed after context switches. **Corresponding panels were added to Figure 3,** illustrating differences between the simple DDM, the Bayesian solution, and our networks in terms of reward rate and adaptation.

Detailed analysis of the neural dynamics and comparison to the DDM.

Beyond behavioral comparisons, we now provide a detailed analysis of how the neural networks implement the solution. Specifically, **we added a new figure (Figure 5)** comparing the dynamics of the full Bayesian model, the DDM, and the network’s internal representations under various conditions. Importantly, we show that the DDM’s dynamics differ qualitatively from the Bayesian model, while the network dynamics more closely resemble joint Bayesian inference rather than disjoint linear estimation as in the DDM. This analysis is presented in the revised Section 3.4, now titled “Neural mechanisms of approximate Bayesian computation.”Our previous PCA analyses complete the picture and reveal how the network encodes the dynamics of both context and state estimates.

We believe these major additions address the principal concerns raised by the reviewers. The revised manuscript is now substantially clearer and provides new insight into how neural networks can learn to perform flexible, approximate Bayesian inference.

List of major revisions

- Added a new subsection (Section 3.4, “Tractable Approximations”) comparing a heuristic linear integrator (DDM-like) model with the optimal Bayesian solution.
- Revised the network analysis (now Section 3.5, “Neural mechanisms of approximate Bayesian computation”) to compare both performance and neural dynamics of the networks with the optimal Bayesian and heuristic models.
- Added a panel to Figure 1 to better illustrate the block structure, addressing Reviewer #3’s comments.
- Added two panels to Figure 4 directly comparing the heuristic model and the Bayesian solution.
- Introduced a new Figure 5, comparing evidence accumulation dynamics across the different models and the network.
- Added further details on Mouse and network training in the Methods section.

Additional, more specific changes are described in the responses to individual reviewer comments below.

Response to Reviewer 1

> The authors conclude that animals perform flexible perceptual inference by inferring contextual statistics as in the normative bayesian model, and that RNNs offer a plausible neural mechanism to implement such computations in biological agents.
>
> Overall, the manuscript is well written and the presentation of results and arguments is very fluid. The main behavioral result, that mice adjust to latent statistics beyond feedback processing, is reminiscent of the seminal work of Gallistel and Gibbon (e.g. Gallistel and Gibbon 2000, Gallistel et al 2001), and well supported by their data.

We thank the reviewer for their thoughtful summary and positive comments. We wish to highlight two crucial clarifications. First, our RNNs were trained using standard reinforcement learning with a small discount factor, not with explicit reward rate maximization. While a low discount factor encourages rapid responses and can increase reward rate, it does not guarantee optimal reward rate.

Second, although the studies by Gallistel et al. are foundational and relevant, there is an important distinction. Those works demonstrate that rats can adapt their expectations after a change in reward statistics, consistent with Bayesian updating. However, they do not show that animals adjust their policy within the first trial following a change, nor do they provide evidence that animals infer context changes directly from sensory inputs. In contrast, our results show that both mice and RNNs learn to flexibly infer context from sensory cues, relying on reward-driven training but not solely on reward feedback.

These points are central to our findings and are now further clarified in the revised Discussion section.

> Drift diffusion models are mentioned at multiple times in the manuscript, and the authors are well aware of their link with optimal perceptual decision making. In the subsection “Comparison with drift-diffusion dynamics” (line 358), the authors verbally compare the normative bayesian solution to so-called “standard DDM assumptions”, i.e. fixed decision boundary and drift rate. However, previous work cited in the manuscript show that normative solutions sometimes correspond to DDMs with collapsing boundaries

(e.g. Tajima et al 2016) and that animal behavior can be accounted for by DDMs with variable and adaptive drift-rates (e.g. Brunton et al 2013). For these reasons, I disagree with the conclusion that "simultaneously inferring both state and context yields distinctive, context-dependent changes in the decision-variable dynamics that break the conventional logic of drift-diffusion models." (lines 392-393).

We thank the reviewer for raising this important point and agree that previous work, such as Tajima et al. (2016) and Brunton et al. (2013), demonstrates that normative solutions can sometimes be framed as DDMs with collapsing boundaries or adaptive drift rates. However, these approaches address the speed-accuracy trade-off, not the joint inference of both state and context, which is the core challenge in our framework. We have now clarified this distinction in the text and have more appropriately referenced Tajima et al. (2016).

Instead of discussing an unfairly restricted version of these models, a quantitative comparison of DDMs simulations with adaptive boundaries and drift-rate would directly test their ability to offer a mechanistic interpretation of behavioral data.

Thank you for the comment. Please See our response above regarding changes to the manuscript.

The authors' use of RNNs is twofold: 1) as an algorithmic implementation of the normative bayesian model, following the idea that drift-diffusion models are not suitable in this case (but see my first point) and 2) as a plausible biological implementation.
They clearly show that a trained RNN converges to approximate the normative bayesian solution in their task at the superficial, behavioral, level, but also at the underlying level of latent decision variables. This result is not surprising given the existing literature on the ability of these models to converge to the optimal solution in inference tasks.

We thank the reviewer for this comment. While it is true that neural networks are powerful and can, in principle, approximate optimal solutions, this outcome is not guaranteed—especially in the context of reinforcement learning, where the optimal solution is never explicitly provided. It is non-trivial that the RNN, trained only with reward-based feedback, learns to infer context from sensory inputs rather than relying solely on rewards. Our results demonstrate that the network develops internal dynamics closely approximating the Bayes-optimal solution, despite the absence of supervised targets. We have now expanded our analysis of these neural dynamics in the revised section, "*Neural mechanisms of approximate Bayesian computation*."

However, I think the authors overstate how these analyses inform us about biological computations for flexible perceptual inference in general.
First, while RNNs can resemble biological neural circuits, the fact that a trained RNN performs near-optimal Bayesian inference in a task does not necessarily make it a biologically plausible mechanism for such inference. This is because, in general, bayesian inference is intractable (even approximate bayesian inference, see Kwisthout et al 2011). Biological systems rely on limited resources, and despite being able to perform computations that mimic normative bayesian inference in some instances, this cannot be guaranteed a priori. For example the statement "while Bayesian inference requires maintaining and updating joint probability distributions over latent variables, the network has discovered an efficient solution that captures the essential dynamics of the decision variable updates." (lines 499-500) does not hold in general, since there is no "efficient solution" for an intractable problem.

We agree with the reviewer that full Bayesian inference is intractable by finite size neural networks, and we mention it in the paper. However, the networks find solutions that: 1) rely on sensory input and

inference and not reward and 2) perform very close to the optimal Bayes limit. We argue that this approximation, or heuristic, is a biologically plausible implementation of optimal Bayesian behavior. We also note that the term “biologically plausible” is widely used in the literature and is often not well-defined. Here, we propose a deterministic neural mechanism that approximates the dynamics of the optimal Bayesian inference, and suggest (only suggest!) that perhaps this is what the brain does.

Similarly, the statement “By training RNNs on such tasks, we uncover neural mechanisms underlying flexible perceptual inference” (line 659) goes too far. Since the RNN reached a near-optimal behavior in this task by using representations similar to those of the normative model, it gives no additional information than the normative model about how biological systems could solve the task, not to mention solving flexible perceptual inference in general.

We rephrased the statement in a more moderate way, and it now says, “*By training RNNs on such tasks, we propose potential neural mechanisms that implement flexible perceptual inference*.”

Second, in order to fully complete the bridge between the normative model and biological system, the authors don’t provide biological neural activity matching that of artificial networks (as in e.g. Sohn et al 2019). This is not a major issue in the scope of their study, but the title is misleading. Moreover, the statement: “This approach bridges theoretical models and experimental neuroscience, offering a powerful framework for studying the mechanisms underlying complex cognitive functions” (lines 660-661) is more a case of wishful thinking than actual demonstration.

We thank the reviewer for this observation. Our work aims to bridge normative models and potential neural implementations, but we agree that directly matching model predictions to biological neural activity would be an important next step. As the reviewer notes, such comparisons are challenging and require dedicated experiments, which we are currently pursuing. We have revised the relevant paragraph in the Discussion to clarify that our work offers a framework and predictions for future experimental validation, rather than a direct demonstration.

Regarding the title, we respectfully maintain that it accurately reflects the scope of the study, which is centered on developing normative models for flexible perception and exploring neural mechanisms that could implement these computations.

Furthermore, while they convincingly show that training the recurrent weights is necessary for matching animal and normative performance, even when explicitly knowing the context, they again underexplain why this is the case. “In contrast, trained recurrent networks (Fig. 5f) develop internal dynamics that independently control both integration rate and baseline in the precise and complementary manner predicted by Bayesian theory.” (lines 598-600) Either the RNN approximates the normative Bayesian model, and therefore offers no additional insight, or it implements a sufficiently close heuristic, which needs to be made explicit (e.g. a dynamically adaptive DDM ?)

We thank the reviewer for highlighting this issue. There are indeed two related but distinct questions here. First, regarding the necessity of trained dynamics: previous studies have shown that Bayesian estimators can sometimes be linearly decoded from random networks, suggesting that much of the computation could be performed at the readout stage. However, as we demonstrate in Section 3.6, training the recurrent weights is critical—not only for reducing trial-by-trial variance at decision time but also for ensuring robust, online Bayesian inference during the operative task. Without training, estimators can be decoded on average but not reliably on individual trials, resulting in poor performance. This could be seen by looking at the readout analysis of the untrained networks (Supplementary figure S.3b).

Second, on whether the RNN implements true Bayesian computation or a heuristic: we do not claim that the network performs exact Bayesian inference, which would be intractable for a large state space. Rather, our analysis shows that the network learns a structured heuristic that goes beyond simple DDM-like integration. This distinction and our new analyses are clarified in Sections 3.3, 3.4, and Figure 5 of the revised manuscript.

To summarize, the authors should deflate their claims about biological plausibility/neural underpinnings (also in the title), and/or give a more in-depth investigation into how neural networks increase our understanding of inference mechanisms with respect to the normative model.

We believe that in the new section and the new figure 5, we show a more in-depth investigation of the neural mechanism behind the approximate, or heuristic, Bayesian solution.

Regarding the claim that flexible perceptual inference is learned, the authors write: "From a computational perspective, our work extends the framework of meta-learning—the concept of learning to learn—to encompass learning to infer." (lines 631-633). This is a strong overstatement since no analysis in the manuscript actually involves learning the adaptive inference mechanisms. The only contribution on this matter is in the behavioral data of the second group (n=7) showing a training effect in mice (naive mice have a lower early contextual adaptation effect than expert mice). While this result is very interesting and indeed suggests that animals learned to infer correctly both the current state and context, all the subsequent analyses involve models with fixed parameters throughout the task. Here, the internals of RNNs might be revealing: since RNNs are not constrained by specific algorithmic assumptions, fitting an RNN to the responses of the animals (and not rewards, see e.g. Eckstein et al 2024) at early and late stages of training could give an insight of how relevant computations are learned.

We thank the reviewer for this important point and agree that our use of "meta-learning" required clarification. In Wang et al, which we cite in the work, meta-learning is defined as "learning to learn": a network is trained to perform online learning, such that it continues to adapt its behavior in dynamic environments through reinforcement learning during task execution, without further synaptic plasticity. In contrast, our results show that, after training, the RNN performs inference on the sensory inputs to detect context. This is a different phenomena as it does not require reward, and it adapts the behavior based on the sensory inputs. We make this comparison as we use the same network model and same training procedure as Wang et al, but we observe different behavior of the trained networks: sensory inference and not reward-based adaptation.

Also, all mice were trained in low uncertainty contexts first (theta = 0.2/0.3). I understand why it's better (and maybe necessary) for the animal to actually learn the task, but it can bias their overall performance (in contrast, the RNNs were trained with all possible contexts). If training with higher initial uncertainty was tested and did not allow the animals to properly learn the task, it should be mentioned.

We thank the reviewer for this observation. It is correct that mice were initially trained in low-uncertainty contexts ($\theta = 0.2/0.3$), as mastering the task at high uncertainty proved too difficult at the outset. This stepwise approach was necessary for successful learning and to establish the association between reward and the safe state, while minimizing Pavlovian bias toward the auditory cue. In contrast, RNNs were trained on all uncertainty levels from the start and could generalize to untrained θ values. We have now clarified in the Methods section that staged training was required for the mice and added more detail about the early training and operant conditioning procedures.

Response to Reviewer 2

The study, which is rigorous, thorough and elegant, describes generic principles relevant for our understanding of this timely and important problem. I congratulate the authors for their accomplishment.

We thank the reviewer for the supportive comment.

The task shares with previous instantiations the informational asymmetry of the two possible observations with respect to the latent state. An essential (and useful) aspect of this asymmetry is that it provides a simple qualitative readout of whether the subject has learned the generative model: integration should be reset with each observation of, in this case, the nogo stimulus (because the prob of observing a nogo in the safe state is zero). **Do mice show evidence of such resetting behavior?** The authors show that waiting times increase with theta, but they do not show whether waiting time is, on average, independent of how many go stimuli were presented before the last nogo.

We thank the reviewer for this insightful comment. Under correct inference, a nogo stimulus should indeed trigger a reset of the decision process, such that only the number of consecutive go cues since the last nogo should influence the decision to act. Go cues preceding the last nogo should not directly affect behavior, except via their impact on the estimate of θ, which would, if anything, decrease the probability of responding.

To directly address this, we performed a GLM regression analysis assessing the effects of go cues both before and after the last nogo on licking probability. The results confirm a resetting strategy: only the number of go cues following the last nogo significantly predicts licking, while go cues before the last nogo have no significant effect. This is consistent with both the Bayes-optimal theory and our trained network.

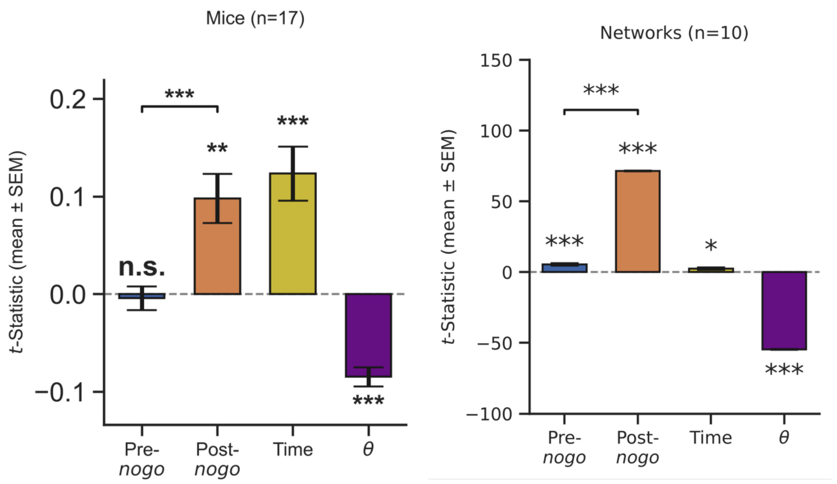

While a deeper analysis of resetting dynamics in mouse behavior is an important topic—one we are currently pursuing—we focused here on context inference and flexible perceptual inference. Nevertheless, we have included the above analysis in the supplementary material.

Is it possible that the mice use a heuristic whereby waiting time is an increasing function of the running average of go stimuli within a trial? In this case, the difference between novice and expert mice might be how deterministic this function is. The optimal strategy can be described in this way, but it would seem

like various suboptimal/heuristic strategies might also have this property. The authors nicely call attention to the difficulty of identifying whether their RNN agents are indeed using the optimal strategy, and it seems like even greater difficulties would apply for real agents where we don't have access to the majority of the relevant internal signals. Along this lines, perhaps the plots suggested in point (1) would provide further evidence that the mice indeed approach the optimal strategy.

We thank the reviewer for this thoughtful suggestion. As noted above, the behavioral effects of go cues depend critically on their position relative to the last nogo—a distinction lost when considering the running average across the trial. Go cues after the last nogo increase the probability of licking by signaling a likely state change, whereas go cues before the last nogo primarily update the estimate of θ, which would decrease responding. This is clearly shown in newly introduced Figure 5.

Our GLM analysis supports an inference-based resetting strategy rather than a simple running average: only the number of consecutive go cues after the last nogo significantly predicts licking behavior. While mice may use the running average to estimate θ, this alone does not account for the observed behavior; their responses clearly depend on the post-nogo sequence, consistent with Bayesian inference.

Theory

The authors employ a different approach from previous works who have used POMDPs for deriving the optimal policy in (different) perceptual decision tasks (e.g., Moreno, Drugowitsch et al., 2012 and ref 12). If I understand correctly, they first compute the optimal waiting time by maximizing the average (over the reliability parameter theta) of an expression for the reward rate conditional on theta. From here, they derive a decision bound by evaluating the average (over the reliability parameter theta) of the GO probability at the optimal waiting time. But the derivation of the optimal waiting time does not depend explicitly on any 'policy'. Only implicitly, I guess, through the fact that the (average) reward rate after the optimal waiting time has been exceeded. I might be wrong, but it doesn't seem trivial to me that the behavior produced by using this policy would be the same as the behavior resulting from a policy that consists in applying, moment by moment a hard bound on the task relevant belief (which is the approach that was followed in previous studies). This is because the belief trajectories in the presence of a bound will not necessarily coincide with those used for computing the various averages in the method used by the authors. Perhaps the authors could clarify this issue.

We thank the reviewer for this perceptive question. Indeed, our approach differs from previous POMDP-based methods. For each context, we compute the optimal waiting time that solves the speed-accuracy tradeoff, which is determined solely by the task parameters (ITI, context, transition probability) and is not derived from an explicit moment-by-moment policy. The primary challenge then becomes inferring the current context, which we treat as a POMDP.

To incorporate the optimal waiting time within the POMDP framework, we translate it into a context-dependent threshold on the state belief. This yields a deterministic policy: wait until the belief crosses the threshold corresponding to the inferred context. Our method allows us to derive a deterministic, context-dependent solution that captures the speed-accuracy tradeoff and provides a clear normative benchmark.

We have clarified these points in Sections 3.3 and 3.4, where we now also explicitly compare hard-threshold and adaptive integration-rate policies.

I think discussing a bit more in detail the similarities/differences between the POMDP solution of the standard 2AFC task in the refs above and the one described here would also be nice.

We explain the similarities and differences above. The new sections in 3.3 and 3.4 now discuss it in more detail.

Are the bounds on belief time dependent? I could not clearly stated whether this is the case except in the discussion in line 614 “bounds dynamically adjust in real time”. It seems to me like they should depend on time because they depend on theta and the agent’s estimate of theta is evolving (at least after a block change), but I might be wrong. This seems quite important so whether they are constant of time-varying should be stated and explained more explicitly.

The decision bounds are not explicitly time-dependent; rather, they are context-dependent and adjust according to the agent’s estimate of the current context (θ). As the context estimate evolves—particularly after a block change—the bound is updated accordingly, but remains fixed within a given context. We have now clarified this in the revised text, and the evolution of context estimates and corresponding bounds is illustrated in Figure 5.

Rigorously speaking, the reward-rate maximizing policy should include, in the denominator the consequences of mistakes where the animal licks in the unsafe state (7 sec penalty). Probably the qualitative features of the solution do not change adding this feature, and thus I don’t think it’s necessary that the authors compute this new policy, but maybe they can comment on this in the discussion.

We thank the reviewer for this overlooked point. In the animal experiments, a 7-second penalty was imposed for licking in the unsafe state, and this was necessary for effective training. In the network simulations, however, this penalty was not required for learning, so it was not implemented or included in our analysis. Had we used such a penalty in the simulations, it could have been readily incorporated by adding the penalty duration weighted by the mistake probability to the denominator of the reward rate. We have clarified this distinction in the Methods section.

I could not fully understand what the cost function was for the networks. In lines 461-62 it says “networks were rewarded for implementing appropriate waiting times”. In the methods (lines 762, 764) the cost is given for the critic, but not explicitly for the actor. Would be nice to clarify.

We thank the reviewer for noting this lack of clarity. The action reward is indeed provided directly to the actor network, following the standard actor-critic framework. We have now added a more detailed explanation of the reward structure and training procedure in the Methods section.

The authors argue that “The network could have adopted simpler strategies, such as basic evidence accumulation with context-dependent thresholds. Instead, it discovered how to approximately mirror the complex, context-dependent dynamics of optimal sequential inference, including precisely calibrated integration rates and initial conditions” (lines 501-504). I’m not sure I understand this reasoning. This seems to suggest that those alternative solutions would be equivalent from the point of view of the cost function? Easier to learn (inductive bias)?

We thank the reviewer for raising this point. We have revised the relevant paragraph in the discussion, which is supported by the added results in Sections 3.3, 3.4, and Figure 5. The network does not simply implement linear evidence accumulation with a context-dependent threshold; instead, it learns a richer internal dynamics that more closely mirrors the full Bayesian solution. This goes beyond what would be expected from a DDM-like strategy and reflects a more sophisticated form of inference.

Response to Reviewer 3

I would like to thank the authors for this nice study tackling a fundamental problem in decision making which how agents flexibly adjust their decisions depending on the context. The authors trained mice to do the a task in which they infer state and context changes which is open up a lot of avenues of behavioral and neural mechanisms.

We thank the reviewer for the supportive comments and for recognizing the significance of our study.

I think the behavior part by itself can be a standalone paper which entails removing the part in which you simulate neural networks generating the neural activity that can underlie the behavioral dynamics you observe…

… the authors can extend the behavioral modeling by simulating the DDM models they are proposing or at least propose a functional form that you think might be underlying the modified DDM model. I would also recommend that you try using a two or multiple accumulator models that can reproduce the behavioral data. We lack in the literature studies that compare POMDPs and DDMs on the same data set, so this might be your opportunity to give us insights into this. Moreover, it will be great to speculate on how can we differentiate between them, may be it is here when the neural mechanisms become crucial.

We thank the reviewer for this thoughtful suggestion. Indeed, the behavioral aspects of our task are rich and merit dedicated analysis; we are currently preparing a complementary manuscript that explores these in detail, including additional behavioral modeling and experimental data. However, we agree with the editor that the core computational questions addressed here—on flexible perceptual inference and its possible neural mechanisms—warrant a focused, unified treatment in the present paper. We therefore reserve more extensive behavioral model fitting, including comparisons to DDM variants and multi-accumulator models, for a future study.

Figure one needs to improved significantly . Fig 1b needs to include the behavioral task design in a more details like in many other mice or rats behavioral papers eg. including the range of the trial length, the values of theta , etc. (I read them later in fig 2B and the Methods but they should be in fig 1 for clarity of the design.) just so that the reader can quickly get the quantitative aspects of the behavioral design.

We thank the reviewer for this suggestion and have redesigned Figure 1 to address several reviewer comments. While we appreciate the desire for more detailed task parameters in the introductory figure, we intentionally kept Figure 1 focused on the abstract and conceptual features of the task to emphasize its relevance for both the experimental and computational components of the paper. Detailed quantitative aspects of the behavioral design are provided in Figure 2B and the Methods, as is customary for primarily theoretical studies. We believe this structure best serves the clarity and narrative flow of the paper.

I think including a psychometric **curve** in fig 1 important to show the overall performance of the mice. It was hard for me to gauge how good they are at doing the task

We thank the reviewer for this suggestion. On average, mice responded correctly in approximately 40% of trials, and this performance was consistent across contexts. However, we note that success rate alone does not capture the speed-accuracy tradeoff that is central to this task; mice could increase their success rate by waiting longer, but this would reduce overall reward rate. It is also unclear how to define chance performance. For these reasons, we focus on reward rate as the primary performance measure and analyze it in detail throughout the paper.

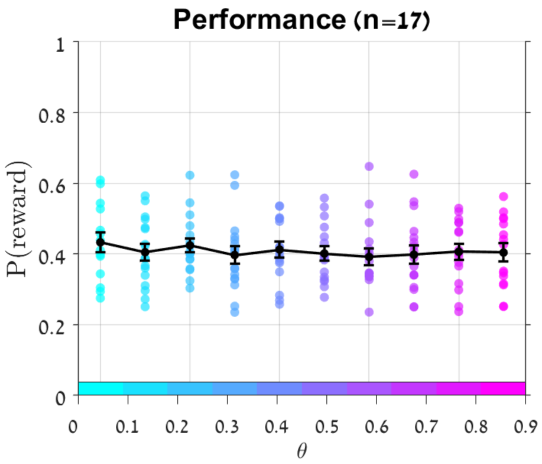

> I understand that mice adapt from the very first trial after switch but i see in fig 2 c that the correlation seems to be increasing so can you clarify the values of what one can call timescale of adaptation. My impression, correct me if I am wrong, is that when the switch happens they are performing the task with okay accuracy then better then better , does the performance plateau afterwards ? …although the switch of the novice is gradual and the expert is quick, to my eyes they reach similar performance after three trials nevertheless they exhibit asymmetry in their behavior during high uncertainty context, what's your interpretation for this ? can this be accounted for using your RL driven RNN training.

We agree with the reviewer's interpretation: steady-state performance after a context switch is similar for novice and expert mice and is typically reached within about three trials. The crucial distinction is in the first post-switch trial, where expert mice already exhibit significantly more adapted behavior. This rapid adjustment cannot be explained by reward feedback, indicating that experts rely more on inference-based strategies rather than simple reinforcement learning. In contrast, novice mice show a gradual adaptation, consistent with greater dependence on feedback. Our RL-trained RNNs also exhibit inference-based adaptation, and we discuss these parallels in the revised Results section.

> I am also curious about the terminology 'safe' or 'unsafe' you can use State 1 and State 2 without using a terminology that might imply that the animal in itself is feeling safe or not (like in a predator - prey context or with looming stimuli).

We appreciate the reviewer's concern about terminology. Our use of 'safe' and 'unsafe' was intentional, as it reflects the core uncertainty in the task—mirroring real-world scenarios like predator-prey contexts. While we acknowledge that terms such as 'state 1' and 'state 2' are more neutral, we found them less descriptive for readers, as they do not convey the underlying meaning of each state as easily.
