# Supplementary material for: Neural mechanisms of flexible perceptual inference
Source: PLoS Comput Biol. 2025 Dec 1;21(12):e1013675. doi: 10.1371/journal.pcbi.1013675 (PMC12680366; doi:10.1371/journal.pcbi.1013675)
Supplement: Attachment — Submitted filename: Second response letter.pdf [file pcbi.1013675.s003.pdf]

We thank the reviewers and the editor for their careful consideration and constructive feedback on our manuscript. We are honored by your positive assessment and your recommendation for publication in PLoS Computational Biology. Below, we provide our responses to the final outstanding comments from Reviewer #2, along with a summary of the corresponding changes made to the manuscript.

Reset at a nogo and finite time step

We thank the reviewer for this important follow-up. Indeed, the apparent lack of an immediate, sharp reset after a nogo cue in our original network analysis is indeed due to the discrete update resolution of the network, not a limitation of the inference strategy itself. To confirm that this is a numerical effect, rather than a fundamental limitation of the network's inference strategy, we trained a network using a timestep five times smaller than in the original simulations (i.e., each input signal was distributed over five network update steps, rather than one).

The attached figure uses the same vector plot format as Figure 5 in the paper: each vector shows the change in the network's projection onto the first two principal components following a nogo cue, with each vector corresponding to a single environmental (input) time bin. As shown, the finer-timestep network (dark red) exhibits a much sharper reset within each environmental step, closely approaching an instantaneous transition, while the original network (light red) displays a more gradual change. This confirms that the gradual reset is solely a result of finite update resolution; with sufficiently small timesteps, the reset becomes effectively instantaneous, limited only by the network's intrinsic dynamics.

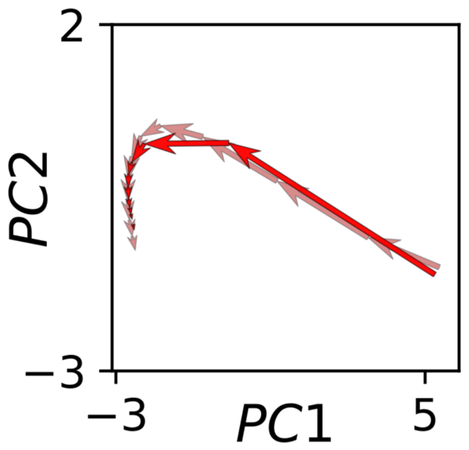

We chose not to include these finer-timestep simulations in the main text for two reasons: first, for consistency, we preferred to keep all analyses based on networks with identical architecture and settings; and second, as discussed previously, the reset dynamics are not the main focus of this paper. Instead, we have clarified in the revised text that this effect is purely numerical and does not reflect a fundamental limitation of the network.

Adding a time course of the decision variable.

We thank the reviewer for this suggestion. We believe there may have been some confusion, as each column in Figure 5 already presents the time-course of the decision variable under identical conditions: the top panel shows the Bayesian theory, the middle panel the simplified model, and the bottom panel the network. Thus, for each set of parameters, readers can directly compare the dynamics of the decision variable and link them with the posterior estimates for state and uncertainty across all three models. We have revised the figure caption to clarify this correspondence.

Summary of changes

- When describing the dynamics of the network in Figure 5, we have added the sentence "*Notably, the networks cannot implement a discontinuous jump, and the sharp reset is implemented in a few steps. This numerical discrepancy disappears when the internal dynamics of the network becomes continuous and is limited only by the internal time constant of the network.*"
- In the caption of Figure 5 we have added "E*ach column show the update dynamics for exact same conditions under the three model with different context values.*"
- We have corrected the typos noted by Reviewer #1
